# HIV rapid test performance among health facilities enrolled in HIV rapid test quality improvement initiative (RTQII) in Ethiopia

**DOI:** 10.1186/s12879-023-08285-x

**Published:** 2023-05-10

**Authors:** Dereje Yenealem, Shemsu Kedir, Asmare MekonnenWubie, Daniel Melese, Tamirat Molalign, Nebiyou Yemanebirhan, Awad Mohammed, Negash Nurahmed, Wondimeneh Liknaw, Abrham Kerealeme Lakew, Habtamu Asrat, Getnet Hailu, Addisu Kebede, Abay Sisay, Kassu Desta, Aster Tsegaye

**Affiliations:** 1 Ethiopian Public Health Institute, Addis Ababa, Ethiopia; 2 Department of Medical Laboratory Sciences, College of Health Sciences, Addis Ababa University, Addis Ababa, Ethiopia

**Keywords:** Acceptable performance, Dried tube specimen, HIV Testing Sites, National HIV Testing Algorithm, External Quality Assessment, Proficiency testing

## Abstract

**Background:**

As the Human Immunodeficiency Virus (HIV) rapid testing services expanded to reach the global target that 95% of people living with the virus will know their status by 2030, ensuring the quality of those services becomes critical. This study was conducted to assess the performance of HIV Rapid testing at sites in health facilities that were enrolled in the Rapid Test Quality Improvement Initiative (RTQII) in Ethiopia.

**Methods:**

Characterized HIV proficiency testing (PT) panels of Dried Tube Specimen (DTS) were prepared, verified, and distributed to testing sites from August to December 2019. In addition on-site evaluation of HIV testing sites (HTSs) was conducted using a checklist to assess testing conditions. For proficiency testing, the study included 159 HIV testing sites (HTSs) in 41 Health facilities (HFs) in five administrative regions and two city administrations. The collected data was analyzed by SPSS version 20 and chi-square test was applied to identify the association between acceptable performance and contributing factors. Testing sites with 100% PT score as well as conducting the test with adherence to the National HIV Testing Algorithm were considered acceptable.

**Results:**

The overall acceptable performance (100% PT score with the correct algorithm followed) was found to be 62% while 12% scored 80% and 11% scored between 20 and 60%. The rest 15% were not considered as acceptable due to failure to adhere to the National HIV Testing Algorithm. Testing sites that participated in External Quality Assessment/Proficiency Testing schemes have shown better performance than those that did not participate with 70% and 56% performance respectively (p = 0.057).

**Supplementary Information:**

The online version contains supplementary material available at 10.1186/s12879-023-08285-x.

## Introduction

The Human Immunodeficiency Virus (HIV) rapid testing services expansion is vital to reach the global target that 95% of people living with the virus will know their status by 2030 [1]. Enhancing the HIV testing accessibility will have significant impact on the prevention, treatment and care of citizens. Accordingly, in Ethiopia the service has been expanded to various testing sites at different health facilities in order to offer many people the chance to be tested. Expanding HIV testing services help in increasing the case detection rate thereby providing information for programmatic interventions [2].However, the effects of this expansion on the quality of testing and the accuracy of test results should be critically monitored.

HIV rapid testing services must ensure that appropriate quality assurance programs are implemented to obtain accurate and reliable test results [3].Proficiency testing programs are important to assessthe HIV rapid testing performance of laboratories and the competence of testing personnel while conducting an onsite evaluation provides a chance to observe the overall testing situation [4].

In view of expanding and improving the quality and safety of HIV rapid testing services, the U.S. President’s Emergency Plan for AIDS Relief (PEPFAR), in 2013 had introduced an HIV Rapid Test Quality Improvement Initiative (RTQII) in seven countries in Africa including Ethiopia. The RTQII is comprised of five key action areas that include: creating enabling environment for policy development, training and certification of testers, monitoring the quality of testing, use of standardized patient registers, and monitoring the quality of test kits and new kit lots procured and used within a given market, program, or country [5]. Consequently, 188 testing sites in 45 hospitals enrolled to bring quality improvement to HIV rapid testing through this initiative in 2015 and 2016 in Ethiopia where initial actions mainly focused on training testers and distribution of standardized registers.

The objective of this study is to assess the performance of HIV Rapid testing sitesat the selected facilities through proficiency testing and onsite evaluation.This study is the first of its kind in trying to evaluate HIV testing Sites (HTSs) performance of HIV Rapid Diagnostic Test (RDT)in a large sample size across Ethiopia.

Proficiency testing (PT) program helps monitor the quality of HIV antibody testing in resource-limited settings. Studies showed that HIV-specific antibodies in the Dried Tube Specimen (DTS) were stable at 4^o^c and 25^o^c for 4 weeks, with only marginal decline at 37^o^c and 45^o^c over 4 weeks. The DTS based PT program was piloted successfully in 24 testing sites in Kenya. And results demonstrated that the DTS is a simple to use, practical method to prepare and distribute PT panels to monitor HIV testing practices in resource-limited settings [6].

**Fig. 1:**
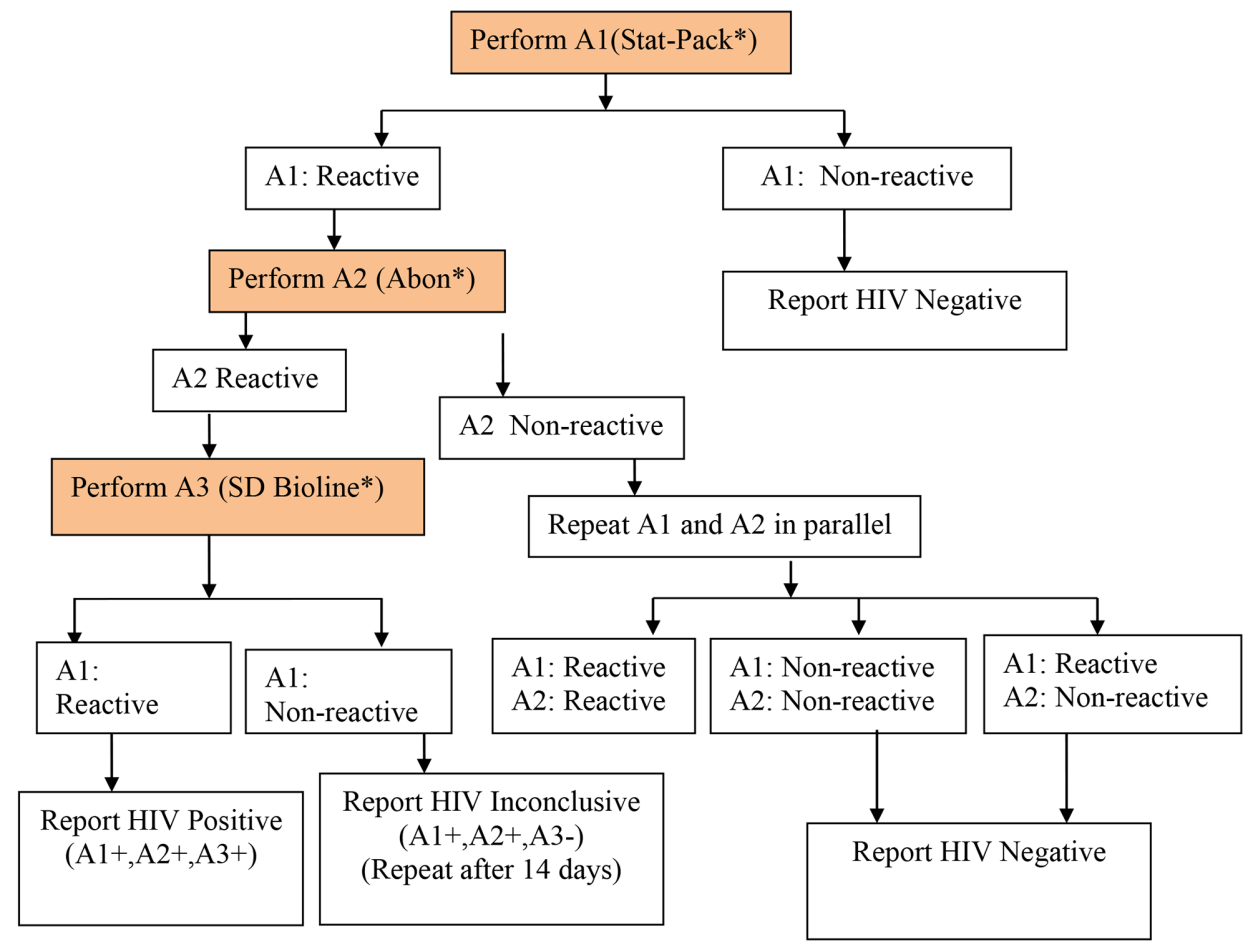
Current National HIV Testing Algorithm (NHTA) in Ethiopia since 2018 [7]. (**Key**: A1 = Assay1, A2 = Assay 2, A3 = Assay 3. * Brand name of HIV rapid test kit)

**Fig. 2:**
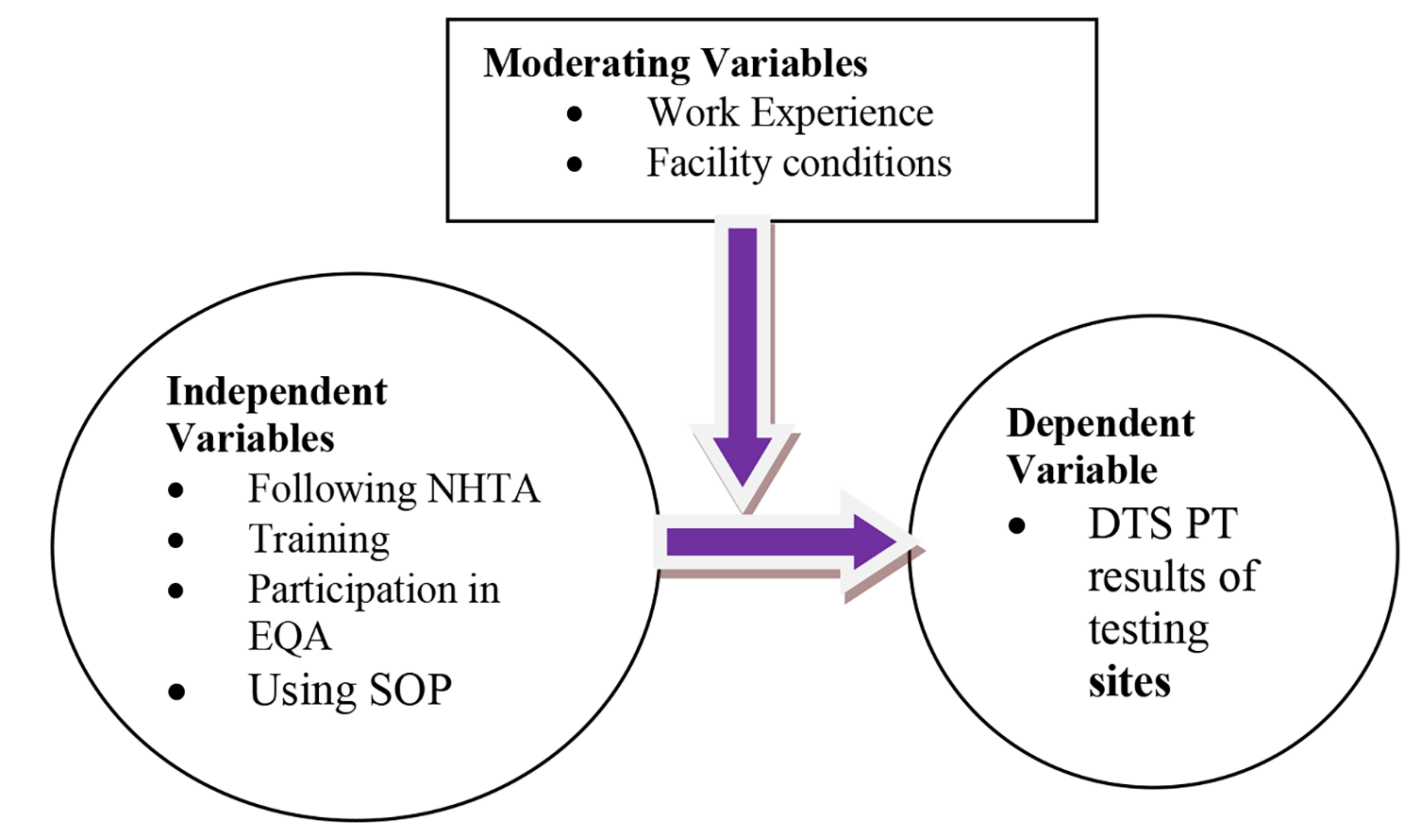
Conceptual Framework

In Ethiopia, the new algorithm, shown in Fig. 1, has been introduced since 2018. This study tries to review HIV rapid test performance in the context of this algorithm and identify the different study variables as represented in the Conceptual Framework- Fig. 2.

## Materials & methods

### Study setting and samples

This study was conducted in five of the nine regions and two City Administrations of Ethiopia. It included a total of 159 HIV Testing Sites (HTSs) in 41 HEALTH Facilities (HFs). The selected sites are HTSs enrolled in the HIV RTQII from five regions (Amhara, Harari, Tigray, Oromia, and SNNPR) and two City Administrations (Addis Ababa, and Dire Dawa). Table 1 shows number of testing sites together with their respective facilities and administrative regions.

**Table 1:** Health Facilities and HIV Testing Sites included in the study

S.#	Region	Number of Health Facilities	Number of HIV testing Sites
1	Addis Ababa	8	27
2	Amhara	9	38
3	Dire Dawa	2	9
4	Harari	2	10
5	Oromia	9	40
6	SNNPR*	5	15
7	Tigray	6	20
Total		41	159

*Southern Nations and Nationalities People Region (at the time of the study)

HIV negative blood samples were obtained from the Ethiopian National Blood Bank (ENBB) based on an agreement concluded between ENBB and the Ethiopian Public Health Institute (EPHI) whereas characterized HIV positive blood specimens were received from CDC-Ethiopia for the preparation of HIV negative and HIV positive PT panels respectively. To each testing site, five, i.e. two positive and three negative HIV DTS Panel samples were distributed. The overall evaluation panel constituted a total of 795 specimens with 318 positives and 477 negatives. DTSs were prepared by drying 20 µl of plasma overnight at a temperature range from 15^o^ to 19^o^ Celsius. The addition of a green dye (0.1%) made the DTS pellets visible without affecting the test results. Phosphate Buffered Saline (PBS) with PH 7.2 was prepared and distributed in separate tubes and sent together with HIV DTS PT samples. Then plasma specimens were reconstituted by adding 7 drops of PBS at or near the testing site and left overnight (12 h). The next day, testing was done following a thorough mixing/tapping of the reconstituted samples.

### Study design

Health facility-based cross-sectional study was conducted from August to December 2019 using non-probability purposive sampling method.

### Data collection procedure

DTS samples were transported to testing sites in leak-proof tubes. Laboratory Technologists with demonstrated skills delivered the DTS panels to the testing sites and conducted on-site assessments using a checklist. The sites were provided with five DTS PT samples (two HIV positives and three HIV negatives) together with buffer solution, pipette, reporting forms, and printed instructions. Report forms containing results of DTS PT samples and completed checklists were submitted to the principal investigator at EPHI who entered the information into the database for analysis. The preliminary data entry were reviewed, and checked through a data cleaning process using the Microsoft Excel program and the Statistical Package for Social Scientist (SPSS) version 20.

### Data quality assurance

In order to ensure homogeneity, a verification test was conducted for 5% of samples from each batch of HIV DTS PT during production time and the status of each coded sample was documented before the DTS PT samples were dispatched to testing sites. Result reporting form and standard checklist were used to collect feedback from testing sites. To maintain consistency and coherence, the principal investigator provided orientation to the data collection team that composed of trained and skilled laboratory technologists.

Stability of the shipped PT materials was monitored by way of sending an additional set of HIV DTS PT samples together with the ones sent to testing sites. These additional samples were sent to undergo all the conditions endured by the HIV DTS PT samples during transportation and made to return back to EPHI to be tested for confirmation that they give the expected results. Thus, all returned samples proved to be stable as they yield the expected results up on return implying that panels received by testing sites had maintained their stability.

### Data interpretation and analysis

Testing sites were considered as having acceptable performance (100% score), when they produce the expected results from the PT panels using the current algorithm. Each of the five panels was given a 20% score totally making up a100% score. Any score less than 100% as well as failure to adhere to the NHTA was considered unacceptable performance. Data collected using checklist was also reviewed to identify factors contributing to acceptable performance by testing facilities. All data were entered in to excel sheet and imported to SPSS version 20 for analysis, cleaned and coded. The contributions of different variables toHIV rapid test performance was evaluated and calculated, differences and agreements between indicators were also assessed. Descriptive and summary statistics were done. Chi-square test was used to determine the association of each independent variable with the outcome variable.

### Operational definitions

#### Dried tube specimen (DTS)

Proficiency testing panels for HIV rapid testing produced by transferring serum or plasma into tubes that are stable at room temperature for at least one month thus can be safely distributed to participants.

#### External quality assessment (EQA)

A system for objectively checking the capabilities of testing sites by an external agency or facility. It allows participants to compare their performance against the expected outcome.EQA includes on-site evaluation, panel testing, and blinded rechecking methods.

#### HIV testing sites (HTSs)

Point of care HIV testing points, not including laboratories, at Health Facilities (Hospitals) that are selected as required by the conditions of health services at respective health institutions.

#### On-Site evaluation (OSE)

An on-site visit to obtain a realistic assessment of the conditions and skills practiced at testing centers. It is usually carried out by experienced personnel from a higher level using a checklist. An on-site evaluation is done as part of an ongoing EQA process.

#### Proficiency testing (PT)

also referred to as Panel Testing, is the process of assessing the competency of testing sites by way of sending panels of well characterized specimens to testing sites for blind testing and evaluating their performances against the expected result.

#### Quality assurance

This is an ongoing set of activities that should be in place during the entire testing process to help ensure that the test results provided are as accurate and reliable as possible.

#### Rapid diagnostic test (RDT)

in vitro diagnostic of immune chromatographic or immune filtration format for the detection of HIV-1/2 antibodies.

#### National HIV testing algorithm (NHTA)

the combination and sequence of specific assaysin HIV testing strategy which is used nationally to diagnose HIV infection, in this case: *STATPAK, ABBON, and SD- BIOLINE.*

## Results

**Fig. 3:**
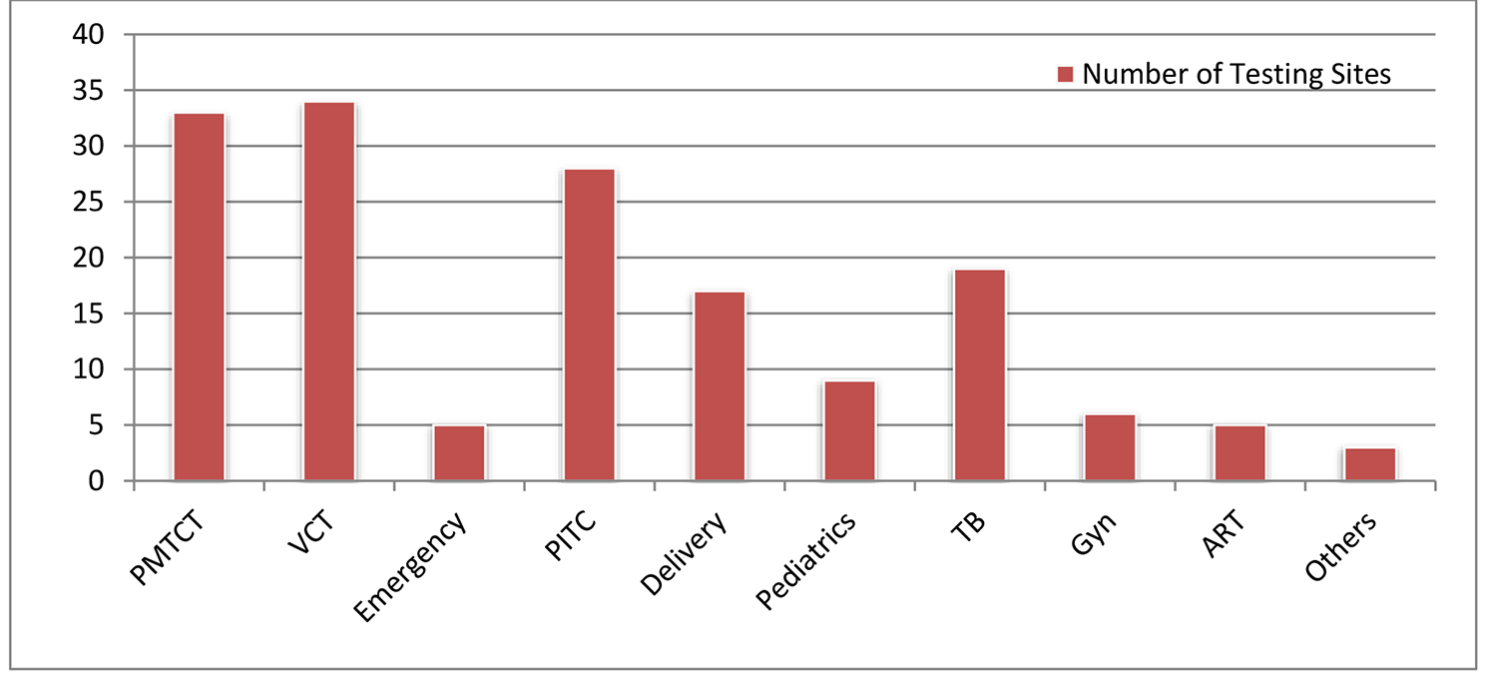
Composition of participant HIV Rapid testing sites (n = 159). (**Key**: Others: OPD Triage, Medical Ward, and Dermatology)

HIV rapid testing sites in public hospitals that were enrolled in RTQII and were providing service at the time of the study were included in this study. Figure 3 shows the names of health facilities and number of the different testing sites under the study. Of the enrolled sites (n = 159), the Voluntary Counseling and Testing (VCT) site was the most frequent (34/159), followed by Prevention of Mother to Child Transmission (PMTCT) site (33/159) and Provider Initiated Testing and Counseling (PITC) site (28/159).

**Fig. 4:**
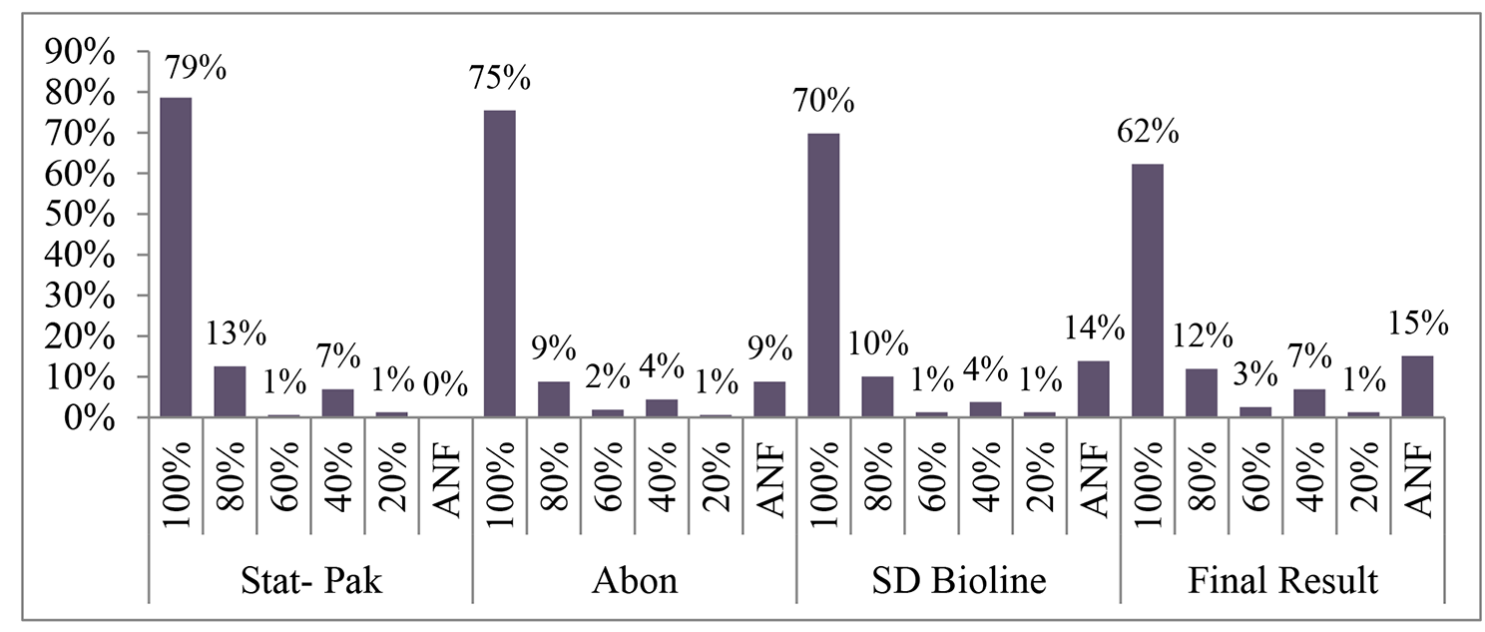
Testing sites’ score (%) for each test kit with final status. (**Key**: ANF: Algorithm Not Followed)

As it can be observed from Fig. 4 below, the overall acceptable performance by HTS was found to be 62% while 12% of HTS scored 80% and 11% of HTS scored between 20 and 60%. Regardless of reported score of the HIV DTS PT samples, HTSs that failed to adhere to the NHTA (15%) were considered as unacceptable.

Assessment of different variables, as shown in Table 2, revealed that almost half (49.7%) of HTSs did not follow SOP, 60% did not have complete testing kits at work stations, 55.3% did not participate in PT in the last two years, 54.4% did not experience supportive supervision from the facility’s laboratory or other higher level agency. With regard to training, 31% (50 out of 159) were not trained on the new NHTA.

**Table 2:** Assessment of different variables at RTQII enrolled HIV testing sites in Ethiopia, August to December 2019 (n = 159)

Variable	Availability		Total
	No	Yes	
Test Algorithm posted	41 (25.8%)	118 (74.2%)	159
Tester followed SOP	79 (49.7%)	80 (50.3%)	159
Trained on the new algorithm	50 (31.4%)	109 (68.6%)	159
All test kits are available at the testing site*	96 (60.4%)	63 (39.6%)	159
Participation in EQA(PT) in the last two years	88 (55.3%)	71 (44.7%)	159
Receive supportive supervision from the facility’s lab	86 (54.4%)	72 (45.6%)	158
Dedicated area(table or bench)for HIV rapid test	57 (35.8%)	102(64.2%)	159
Sink with water supply in the testing area	56(35.2%)	103(64.8%)	159
Disinfectant for decontamination	49(30.8%)	110(69.2%)	159
Proper disposal of biological waste	38(23.9%)	121(76.1%)	159
Sharps container at testing site	22(13.8%)	137(86.2%)	159

*Key: HTSs most of the times keep only one or two of the three HIV test kits at work stations

**Fig. 5:**
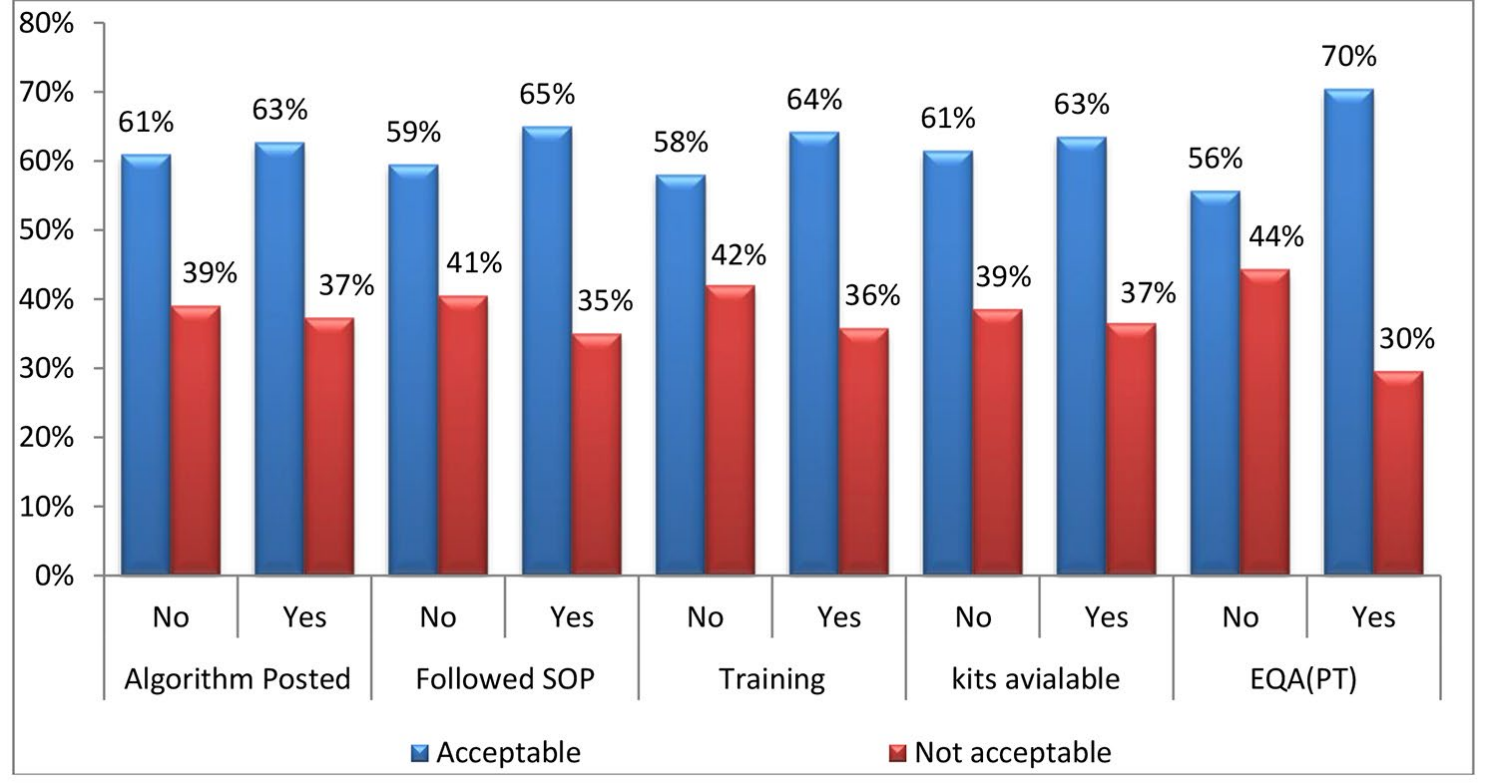
Contribution of variables on testing performance

According to a chi-square test of association between HIV testing performance by testing sites and selected indicators, as indicated in Table 3, participation in EQA/PT panels has shown a close association with acceptable performance with marginal significance ( p = 0.057). Those which participated in PT showed a better performance than those that did not participate in PT schemes with 70% and 56% acceptable performance respectively. Though adherence to the NHTA, Training on HIV rapid testing, following SOP, were all positively associated with HIV rapid testing performances as shown also in Fig. 5, they were not found to be statistically significant.

**Table 3:** Result of Chi-square test of association between HIV testing performance and associated factors by testing sites in Ethiopia, August to December 2019 (n = 159)

Indicators	Values	Performance Status				Total	Chi-square	P-value
		Acceptable		Not Acceptable				
IQC Conducted	No	52	38.50%	83	61.50%	135	0.233	0.629
	Yes	8	33.30%	16	66.70%	24		
Tester followed SOP	No	47	59.50%	32	40.50%	79	0.513	0.474
	Yes	52	65.00%	28	35.00%	80		
Trained on the new algorithm	No	29	58.00%	21	42.00%	50	0.564	0.452
	Yes	70	64.20%	39	35.80%	109		
Experience in years	< 2	25	61.00%	16	39.00%	41	1.451	0.484
	2–5	47	58.80%	33	41.30%	80		
	> 5	26	70.30%	11	29.70%	37		
All test kits available at testing site	No	59	61.50%	37	38.50%	96	0.067	0.796
	Yes	40	63.50%	23	36.50%	63		
Difficulty of the new algorithm compared with the old one	More difficult	65	61.30%	41	38.70%	106	3.928	0.269
	Comparable with previous one	14	70.00%	6	30.00%	20		
	Less difficult	11	78.60%	3	21.40%	14		
	Don’t know	9	47.40%	10	52.60%	19		
Participation in EQA(PT) in the last two years	No	49	55.70%	39	44.30%	88	3.634	0.057*
	Yes	50	70.40%	21	29.60%	71		
Receive supportive supervision from the facility’s lab	No	52	60.50%	34	39.50%	86	0.195	0.659
	Yes	46	63.90%	26	36.10%	72		

*Key: marginally significant variable (P ≤ 0.1)

## Discussion

This study was conducted to assess the performance of HIV Rapid testing sites enrolled in Rapid Test Quality Improvement Initiative (RTQII)through proficiency testing and onsite evaluation in the context of the current National HIV Testing Algorithm (NHTA). In this study, non adherence to the NHTA refers to failure to perform a second test and a third test for a reactive result with the first kit, or reporting a result from the second kit and/or third kit while first test kit result of the PT sample proved to be non reactive. In relation to assessment of proficiency testing results of participant HIV rapid testing sites (HTS), acceptable performance i.e. 100% PT score with the correct algorithm followed, was 62% while 12% of HTS scored 80% and 11% of HTS scored between 20 and 60%.

HIV PT programme conducted from 2006 to 2017 in Haiti showed an average > 90% HIV RDTs score across laboratories, which is much higher than the current study. However, capturing the quality status of routine HIV RDT in the field was the challenge and limitation of the PT programme since HIV RDTs are performed not only by laboratory technicians but also by nurses and aid-nurses that are usually not trained or have received limited on-the-job training [2].In this regard, a similar concern needs to be taken in to consideration for the case of Ethiopia where HIV testing services has been taken out of the laboratory.

Despite a correct final report of the HIV DTS PT samples, 15% were not considered as acceptable due to failure to adhere to the NHTA. This finding is comparable to different studies conducted in Addis Ababa and Tigray region, Ethiopia which revealed a 10% and 12% failure to follow the NHTA respectively [8, 9] while another study in Kenya showed 12.9% non-adherence to the NHTA [10].

Comparison of performance among testing sites indicated that VCT showed better performance, i.e. 73.5% acceptable, seconded by PMTCT 66.7%, then PICT 60.7%, Delivery (52.9), and TB clinic 52.6%, others having a lower score. With regard to training, 31% (50 out of 159) of testers were not trained on the new NHTA. According to a study conducted at point of care sites in Addis Ababa, 56% coverage of HIV training was indicated among testers [8], a finding which is lower than our study. This is of concern as it is demonstrated in the current study the majority of testers who obtained unacceptable result were those who had not been trained. Only 36% of testers with unacceptable results actually got training signifying the need for on the job refreshment trainings or continuing professional development (CPD). The current initiative in Ethiopia that makes CPD mandatory for health professionals will have a positive impact regarding such gaps.

In our study participation of EQA was very low (44.7%, 71/159). It was evident that participation was even much lower in those obtaining unacceptable result in the current audit. A study conducted in 2016 among RTQII sites for rapid testing in Tanzania used seven standards for auditing performance of testing sites. The standards include personnel training and certification, physical facility, safety, pre-testing phase, testing phase, post testing phase, and external quality assurance (EQA) through proficiency testing (PT). Across the seven quality standards, none of the facilities performed at an expected level. The performance gaps were found to be much wider for personnel training and certification (81%), EQA (70%), testing phase (61%) and safety (50%) [11]. In fact, from chi-square test of association, the most important indicator that is associated with acceptable performance in our study was participation in EQA (PT) with marginal significance of p = 0.057(p ≤ 0.1).

These findings underscore the need for well-established EQA with appropriate supervision and trainings based on identified gaps. Over half (54.4%) of the testers in our study did not receive supportive supervision from the facility’s main laboratory. This is a clear message that where task shifting is excercised due to unavoidable circumstances, testers should get appropriate training as well as regular supervision from the main laboratory.

The objective assessment result that 64% of those who got supportive supervision versus about 36% of those with no suppervision scored acceptable result again emphasizes the need for due considerations of EQA to any testers in general and during task shifting in particular to ensure quality HIV rapid testing and scaling up of the HIV testing service to meet the first 95% goal of UNAIDS by 2030 [1].On the other hand, in relation to facility conditions, 36% of testing sites did not have dedicated area (table/bench) for conducting HIV rapid test, 31% were without disinfectant for decontamination, 35% had no clean water supply for hand washing, while 14% did not have sharps container to dispose of sharps. These finding seem to be comparable with findings conducted on 145 testing sites in Tigray which indicated that 41% of testing sites had no designated area for conducting HIV rapid test and 40% had no clean water for hand washing [9].

Finally, although this study was conducted during the end of 2019 and no recent audit is undertaken, it provides valid information to the current situation due to the fact that there has not been major interventions taken so far towards improving HIV rapid testing services at testing sites up to the present day. It is also true that much attention with large proportion of resources has been shifted to the COVID-19 pandemicin the last few years, attributable to the possible catastrophic impact of the disease on public health at large.Now, as the circumstances seem to have changed, it may be time to give attention to improvement areas across other health services including implementing action plans to resolve identified problems from the findings of this study.

## Limitation

This study did not provide detailed information about the types and proportions of errors such as false positive and false negative results. Some testing sites lacked one or two of the three test kits to complete the algorithm. Thus, they were discredited as producing unacceptable performance which likely contributed to lowered overall performance by participant testing sites.

## Conclusion and recommendation

Having in mind the fact that this study discredited “correct” reports that were produced by using non standard procedures which are not in compliance with the NHTA, the overall acceptable HIV rapid testing performance by HTSs was still generally low. In relation to physical facility and organization,for example having designated area for testing, availability of working bench, sink with water for hand washing, adequate supply of test kits and other necessary supplies, improvement is needed to ensure smooth HIV rapid testing service at the testing sites.Facilities that were selected for Rapid Test Quality Improvement Initiative (RTQII) program of HIV RDT had received limited support from PEPFAR implementing partners including higher governmental health institutions. The support was not only short lived but also fall short of bringing quality improvement in the area.

The expansion of HIV testing services at different points of care must ensure that quality assurance programs, especially EQA/PT are employed. In addition, training on the NHTA, regular follow up, assignment of competent personnel, good supply management of HIV test kits together with organized workstation setup are some of the key improvement activities that need to be undertaken in order to provide quality service with regard to HIV RDTs.Therefore in order to resolve the problem, action plans need to be set and implemented to ensure a continuous EQA-PT program that enables testing sites receive HIV PT panels. This should be followed by monitoring activities that includes conducting objective gap assessment and taking effective corrective actions. Future directions with regard to creation of testing sites in a given facility should be based on need analysis, availablity of trained personnel, test kits and other resources. In some cases reducing the number of testing sites may help minimize chances for provision of sub standard services. Assignment of testing personnel should focus on retention mechanisms to decrease frequency of turnovers and manage shifts.

## Electronic supplementary material

Below is the link to the electronic supplementary material.

Supplementary Material 1

Supplementary Material 2

Supplementary Material 3

Supplementary Material 4

Supplementary Material 5

Supplementary Material 6

Supplementary Material 7

Supplementary Material 8

Supplementary Material 9

Supplementary Material 10

Supplementary Material 11

## Data Availability

All data generated or analyzed in during the study are available in this published article and its supplementary information files.
