# Supplementary material for: HIV rapid test performance among health facilities enrolled in HIV rapid test quality improvement initiative (RTQII) in Ethiopia
Source: BMC Infect Dis. 2023 May 10;23:315. doi: 10.1186/s12879-023-08285-x (PMC10170438; doi:10.1186/s12879-023-08285-x)
Supplement: Supplementary file 1 — Supplementary Material 1 [file 12879_2023_8285_MOESM1_ESM.docx]

**Instruction for HIV DTS Panel test specimen re-constitution**

**Scheme description**: **HIV DTS Panel test**

**Scope and Objective of the PT scheme:**

This PT assessment helps strengthen quality improvement in HIV rapid testing sites and also serves as an input for taking corrective action where necessary.

**Package contains**: Five DTS samples and one PBS buffer with in cryo vials randomly labeled with DTS 1, DTS 2, DTS 3 DTS 4, DTS 5 and PBS

**Temperature**: The PT items are shipped and transported at ambient temperature. They should also be processed at ambient conditions.

**Method/tests to be used**: test using current national HIV testing algorithm

**Source of PT items:** characterized samples were used for DTS PT preparation

**Safety requirements:** use universal safety precaution to handle the DTS sample

**DTS Re-constitution procedure**

- - 1. Tap the tube gently before opening the cap to ensure that the colored pellet falls to the bottom of the tube.
    2. Dried tube specimen is re-constituted one day prior to testing.
    3. Using the dropper provided, add 7 drops of PT buffer (equal to 200 μL) to each DTS to be tested. Cover the tube, tap gently and incubate for minimum of 2 hours for same day testing, or overnight at room temperature for next day testing.
    4. Before testing mix the specimen by gently tapping the tube.
    5. Test the re-constituted DTS with HIV rapid test kit as that of you do for patient specimen testing.
    6. Report the results using report form

For any information or details, you can communicate with:

**Dereje Yenealem, Mobile Phone +251 911 425553**
