# Supplementary figures and images for: HIV rapid test performance among health facilities enrolled in HIV rapid test quality improvement initiative (RTQII) in Ethiopia
Source: BMC Infect Dis. 2023 May 10;23:315. doi: 10.1186/s12879-023-08285-x (PMC10170438; doi:10.1186/s12879-023-08285-x)

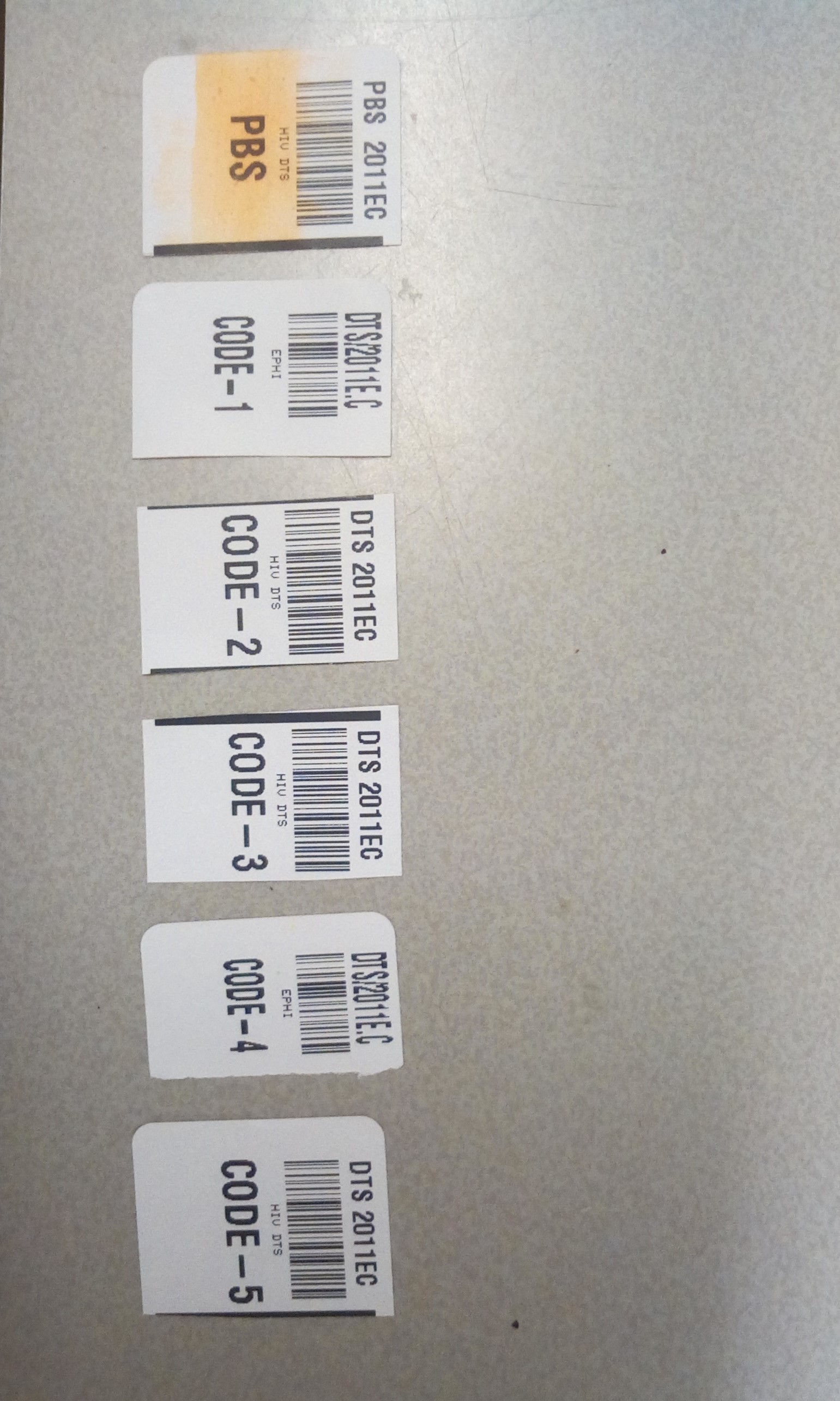

Supplement: Supplementary file 2 — Supplementary Material 2 [file 12879_2023_8285_MOESM2_ESM.jpg]

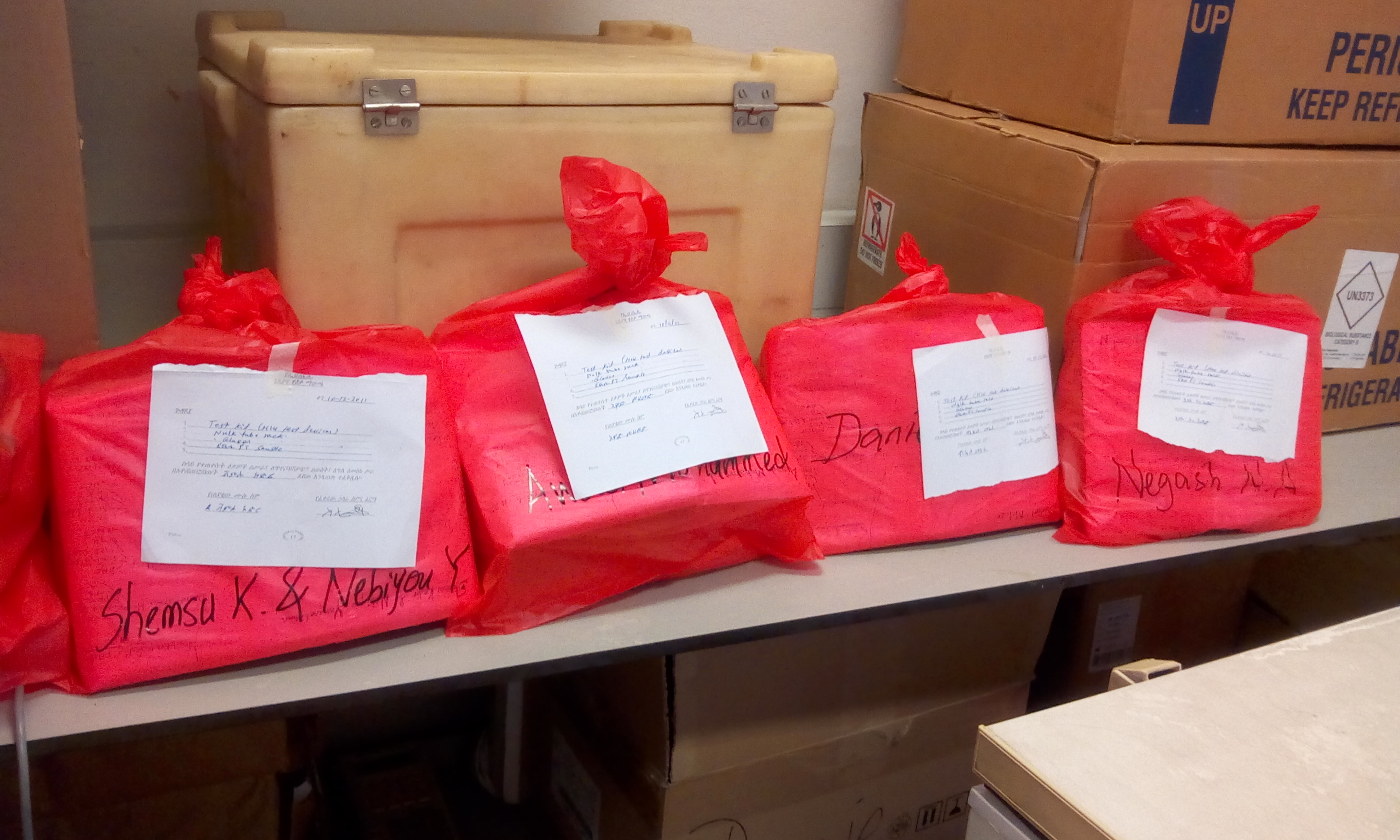

Supplement: Supplementary file 3 — Supplementary Material 3 [file 12879_2023_8285_MOESM3_ESM.jpg]

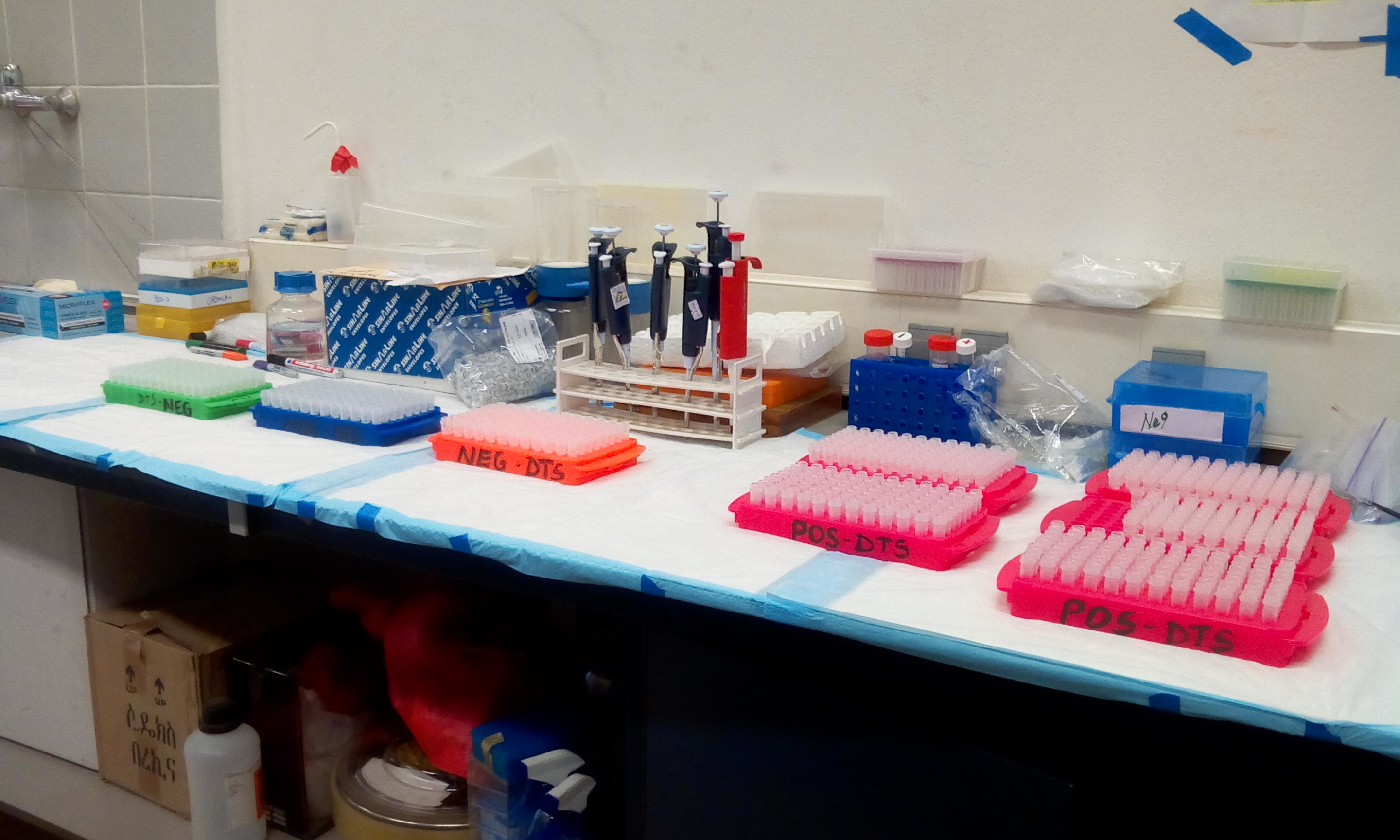

Supplement: Supplementary file 4 — Supplementary Material 4 [file 12879_2023_8285_MOESM4_ESM.jpg]

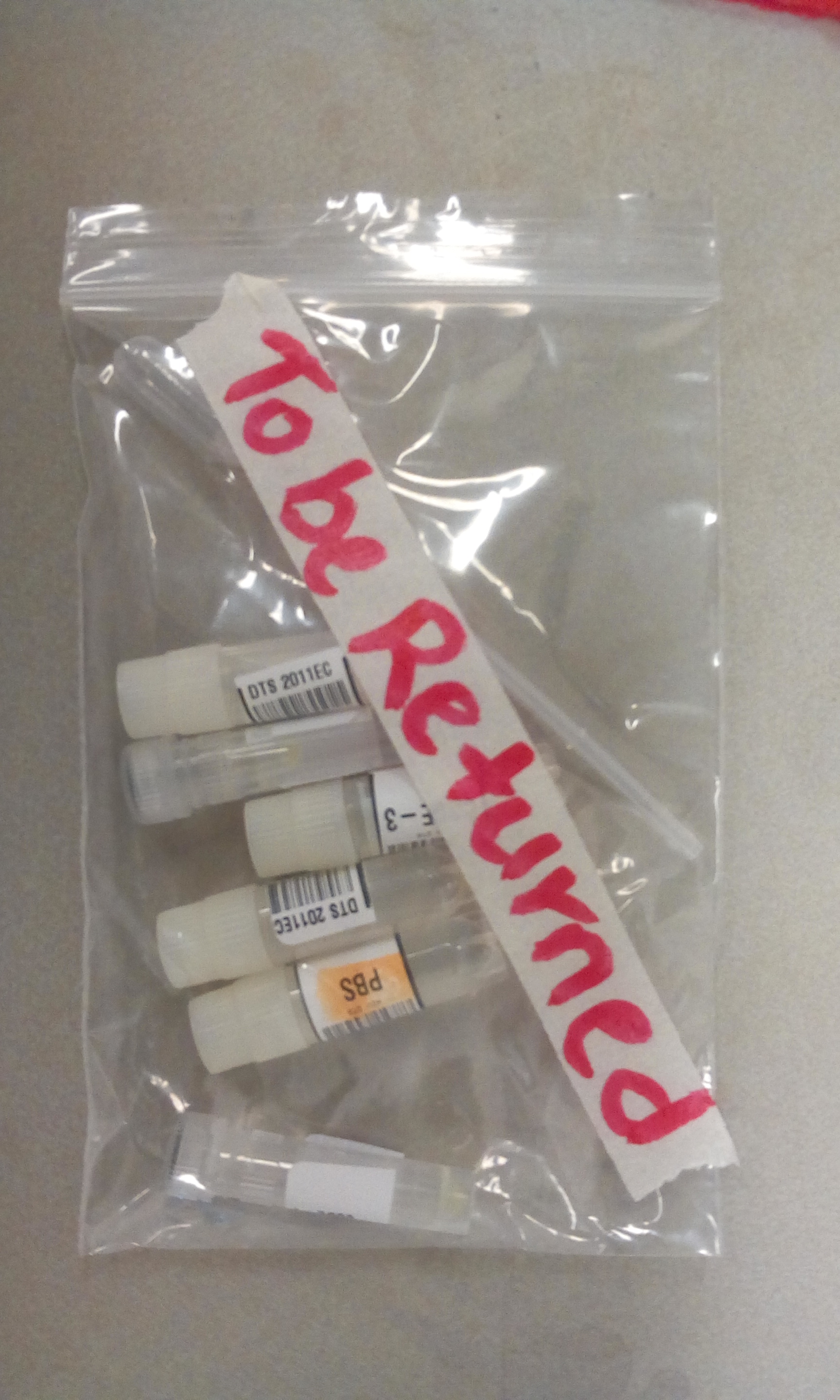

Supplement: Supplementary file 5 — Supplementary Material 5 [file 12879_2023_8285_MOESM5_ESM.jpg]

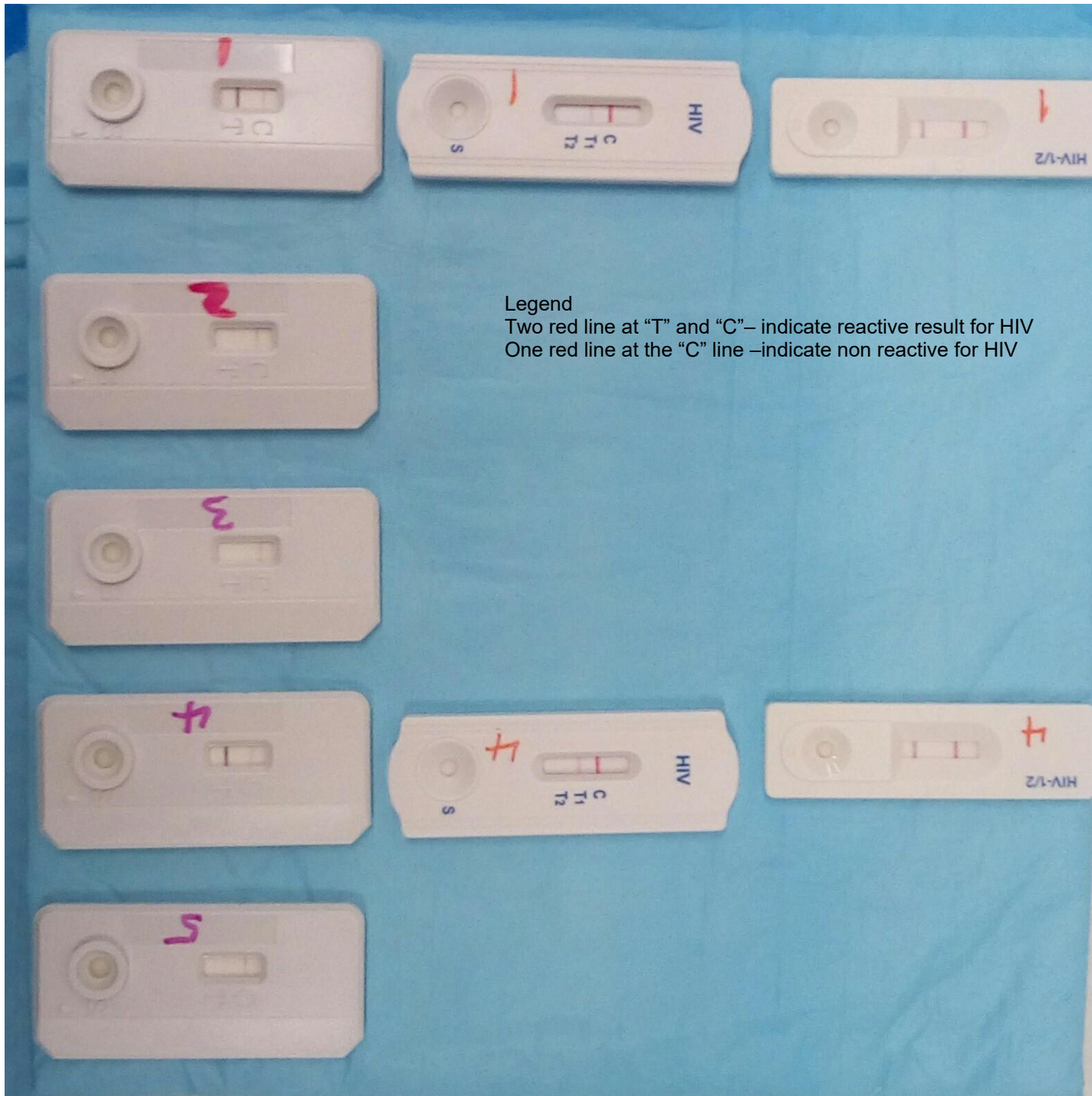

Supplement: Supplementary file 6 — Supplementary Material 6 [file 12879_2023_8285_MOESM6_ESM.pdf]
